# Development of oligomannose-coated liposome-based nasal vaccine against human parainfluenza virus type 3

**DOI:** 10.3389/fmicb.2013.00346

**Published:** 2013-11-26

**Authors:** Kyosuke Senchi, Satoko Matsunaga, Hideki Hasegawa, Hirokazu Kimura, Akihide Ryo

**Affiliations:** ^1^Department of Microbiology, Yokohama City University School of Medicine, Yokohama, KanagawaJapan; ^2^Department of Pathology, National Institute of Infectious DiseasesTokyo, Japan; ^3^Infectious Disease Surveillance Center, National Institute of Infectious DiseasesTokyo, Japan

**Keywords:** HPIV3, HN, vaccine, oligomannose-coated liposome, adjuvant

## Abstract

Human parainfluenza viruses (HPIVs) are the etiologic agents of lower respiratory infections and pneumonia in infants, young children and immunocompromised hosts. The overarching goal for the prevention of HPIV infection is the development of an effective vaccine against HPIVs. In the present study, we investigated the effectiveness of oligomannose-coated liposomes (OMLs) as an antigen-delivery system in combination with a synthetic double-stranded RNA analog for the induction of mucosal and systematic immunity against HPIV3. Full-length hemagglutinin-neuraminidase (HN) protein was synthesized using the wheat germ cell-free protein production system and then encapsulated into OML to serve as the antigen. Intranasal administration of the HN-filling OML (OML-HN) with the synthetic double-stranded RNA adjuvant, polyriboinosinic-polyribocytidylic acid [poly(I:C)] generated significant viral-specific systemic and mucosal immune responses as evidenced by the prominent induction of serum IgG and nasal wash IgA, respectively. On the other hand, no significant immune responses were observed in mice immunized with OML-HN without the adjuvant. Furthermore, serum from mice immunized with OML-HN plus poly(I:C) significantly suppressed viral infection in cell culture model. Our results provide the first evidence that intranasal co-administration of OML-encapsulated HN with the poly(I:C) adjuvant augments the viral-specific immunity against HPIV3.

## INTRODUCTION

Human parainfluenza viruses (HPIVs) belong to the Paramyxoviridae family and are one of the major causes of acute respiratory infections (ARIs) and asthma in infants and young children (<5 years old). HPIVs were classified into four serotypes including HPIV1-4 (Henrickson, 2003; Mizuta et al., 2011). In particular, human parainfluenza virus type 3 (HPIV3) is an important infectious agent, second only to respiratory syncytial virus (RSV), that causes bronchiolitis and pneumonia in infants (Glezen et al., 1984; Counihan et al., 2001; Belshe et al., 2004; Schmidt, 2011). Therefore, the development of a practical vaccine that can prohibit HPIV3 infection in infants is urgently needed.

Currently, there is no prophylactic human vaccine against HPIV3 infection. Several previous studies employed attenuated viruses or recombinant viruses for vaccination by intranasal administration (Haller et al., 2000; Karron et al., 2011; Schmidt et al., 2011; Mason et al., 2013). The HPIV3 cp45 is a practical nasal vaccine that is derived from the JS wild-type strain of HPIV3 through 45 passages in African green monkey cells at a low temperature. This vaccine has been evaluated in clinical human trials and is known to induce the hemagglutination-inhibiting (HAI) antibody in seronegative children (Skiadopoulos et al., 1999; Karron et al., 2003; Belshe et al., 2004). The rB/HPIV3b vaccine is a cDNA-derived chimeric HPIV3 in which the genomic cDNA is partially recombined with bovine PIV3 (BPIV3); the hemagglutinin-neuraminidase (HN) and F genes from HPIV3 fused with BPIV3 whole genome (Schmidt et al., 2001; Karron et al., 2012). The rB/HPIV3 vaccine was shown to induce significantly higher titers of HAI antibodies against HPIV3 in seronegative children. A major limitation of these vaccines is their potential to cause actual infection diseases in children or immunocompromised hosts because they are live attenuated vaccines. Therefore, it is necessary to develop a safer HPIV3 vaccine with lower risks for infection that will be useful for infants and young children in clinics. In this regard, component vaccines are desirable because they use non-infectious viral subunit proteins as antigens. A previous report demonstrated the efficacy of subunit vaccines that target the HPIV3 HN and F proteins in an animal model (Ray et al., 1988). Other reports also demonstrated the induction of protective antibodies that prohibit HPIV3 infection in response to subunit vaccines that target HPIV3 antigens (Morein et al., 1983; Ray et al., 1985; Ambrose et al., 1991; Brideau et al., 1993). A caveat of subunit-based vaccination strategies is their requirement for large amounts of antigens, thus rendering them costly to produce. Therefore, it is important to develop an effective subunit vaccine that utilizes lower quantities of antigen.

To circumvent the aforementioned problems, oligomannose-coated liposome (OML) was used as a natural and non-toxic antigen-delivery system. OML efficiently targets proteins to antigen presenting cells (APCs), such as macrophages or dendritic cells (Shimizu et al., 2007; Nishimura et al., 2013). Furthermore, previous reports showed that antigens incorporated into OML were efficiently delivered to APCs by intranasal administration (de Haan et al., 1995; Ishii and Kojima, 2010; Giddam et al., 2012). The effect of OML was shown to be relatively ineffective at inducing humoral immunity, while it preferentially activated cell-mediated immunity via cytotoxic T lymphocytes (CTLs). Therefore, for optimal induction of both humoral and mucosal immunity it is necessary to use vaccination strategies that combine OML with other adjuvant systems.

Herein, we sought to utilize OML in combination with an adjuvant double-stranded RNA polyinosinic-polycytidylic acid [Poly(I:C)] for the induction of effective humoral and mucosal immunity against HPIV3. The overarching goal was to establish a vaccination strategy that required a small amount of antigen and a few doses. Poly(I:C) is an effective adjuvant for antibody and multi-functional CD4+ T cell responses against viral infection. Poly(I:C) was shown to be an effective mucosal adjuvant for the development of antigen-specific immunity even when hosts were immunized with a relatively small quantity of antigen (Ichinohe et al., 2005; Hasegawa et al., 2009). In addition, we also took advantage of the wheat germ cell-free protein production system to synthesize our antigen, full-length HPIV3-HN protein (Takai et al., 2010). Our results highlight the utility of combining sophisticated systems in the development of a novel vaccine against HPIV3.

## MATERIALS AND METHODS

### CONSTRUCTION OF WHEAT CELL-FREE EXPRESSION VECTOR

HPIV3 (C243) cDNA was kindly provided by Dr. Tsukakoshi. The HN fragment was amplified by PCR using the primers BamHI-HN F (5′-GAGAGGATCCCATGGAATACTGGAAGCAT) and NotI-HN R (5′-GAGAGCGGCCGCTTAACTGCAGCTTTTTGGA). The amplified fragment was digested with BamHI and NotI and cloned into either pEU-His or pEU-GST vectors that were previously digested with the same enzymes. GST-tagged HPIV3-HN (GST-HN) construct was mutated using the reagents of a PrimeSTAR Mutagenesis Basal Kit (TakaraBio, Otsu, Japan) according to the manufacturer’s instructions.

### CELL-FREE PROTEIN SYNTHESIS AND PURIFICATION

In vitro transcription and cell-free protein synthesis were performed as described (Takai et al., 2010). The bilayer translation method was used to synthesize His-tagged HPIV3-HN (His-HN) protein using wheat germ extract that was optimized for Ni-affinity purification (WEPRO 7240H; Cellfree Sciences, Yokohama, Japan) and a robotic synthesizer (Protemist XE; Cellfree Sciences) according to the manufacturer’s instructions. The cell-free translation reaction (15 ml) was separated into soluble and insoluble fractions by centrifuged at 15000 rpm for 15 min. The insoluble fraction was lysed using 8M Urea at room temperature for 6 h and then mixed with Ni-sepharose High Permormance beads (GE Helthcare, Hino, Japan) in the presence of 20 mM imidazole. The beads were washed three times with washing buffer (20 mM Tris–HCl, pH 7.5, 500 mM NaCl) containing 40 mM imidazole. The His-HN protein was then eluted using washing buffer containing 8M Urea, 500 mM imidazole. Purified His-HN proteins were concentrated approximately 10–20-fold using Amicon Ultra Centrifugal Filters (Merck Millipore, Billerica, MA, USA). Full-length GST-HN protein and GST-HN deletion mutant proteins synthesized using wheat germ extract optimized for GST-affinity purification (WEPRO 1240G; Cellfree Science) according to the manufacturer’s instructions. Quantification of synthesized proteins was performed by densitometric scanning of the Coomassie Brilliant Blue^®^ (CBB)-stained bands.

### PREPARATION OF LIPOSOMES

Liposomes were prepared as described previously (Giddam et al., 2012; Nishimura et al., 2013). Briefly, a chloroform:methanol (2:1, *v*/*v*) solution containing 1.5 μmol of DPPC, 1.5 μmol of cholesterol and varying amounts of Man3-DPPE (0.15–0.0015 μmol) was added to a flask and evaporated to prepare a lipid film. PBS containing 1 or 0.5 mg/ml of full-length HPIV3-HN protein was added to the dried lipid film and multi-lamellar vesicles were prepared by intense vortex dispersion. The vesicles were extruded 10 times through a 1 μm pore polycarbonate membrane (Nucleopore, Pleasanton, CA, USA). The amount of entrapped protein was measured using a Modified Lowry Protein Assay Kit (Pierce, Rockford, IL, USA) in the presence of 0.3% (*w*/*v*) sodium dodecyl sulfate using HN as the standard. The particle size of the liposomes was determined using a dynamic light scattering particle size analyzer (BioMedCore Inc., Yokohama, Japan).

### IMMUNIZATION OF MICE

Female BALB/c mice (Japan SLC Inc., Hamamatsu, Japan), age 6–8 weeks at the time of immunization, were used in all of the experiments. All animal experiments were carried out in accordance with the Guides for Animal Experiments Performed at Yokohama City University (YCU) and approved by the International Animal Care and Use Committee of YCU. Three to six mice for each experimental group were anesthetized with isoflurane prior to being immunized. For the primary immunization, 13 μl of single-shot mixtures were prepared as containing OML-HN (0.1 or 1.0 μg) and/or poly(I:C) (10 μg), and administered 6.5 μl of mixtures into each nostril. Three weeks later, the secondary immunization was administered in the same manner. Two weeks after the secondary immunization, as tertiary immunization, 16 μl single-shot mixtures were prepared as containing OML-HN (0.2 or 2.0 μg) and/or Poly(I:C) (10 μg), and administered 8.0 μl of mixtures into each nostril.

### ENZYME-LINKED IMMUNOSORBENT ASSAY

Serum was collected on days 7 and 14 after the secondary immunization and on days 7, 14, 21, and 28 after the tertiary immunization. On day 28 after the third immunization, all of the mice were sacrificed and nasal wash fluid was collected by washing the nasal cavity of the excised head with 1 ml of PBS(-) containing 0.1% BSA. The levels of IgG and IgA antibodies against HPIV3-HN in the serum and nasal wash fluid were determined by enzyme-linked immunosorbent assay (ELISA) as described previously. Briefly, ELISA was conducted sequentially from the solid phase (Anti-GST coated 96-well plate; Thermo, Waltham, USA) with a ladder of reagents consisting of the following: (1) GST-HN protein and GST protein as a control; (2) serum or nasal wash fluid; (3) either anti-mouse IgG antibody-conjugated HRP (1:10000, Thermo) or anti-mouse IgA antibody-conjugated HRP (1:10000, BETHYL, Montgomery, TX, USA); (4) TMB substrate buffer (Thermo); and (5) 2M sulfuric acid. The chromogen produced was measured by determining the absorbance at 450 nm with an ELISA reader. The relative levels of IgG and IgA antibodies against HN were determined relative values calculated as follows; Relative values = mean value in immunized vaccine group/mean value in immunized OML-empty group. Each values were normalized with the optic values to GST protein.

### IMMUNOBLOTTING

Using standard immunoblotting methods, the presence of HN-specific IgG was detected using pooled serum from each group of mice and incubated with anti-mouse IgG HRP-conjugated secondary Ab (Thrmo) at a dilution of 1:10000 in TBST. Immobilon was used for detection (Merck Millipore).

### QUANTITATIVE REAL-TIME PCR

We performed an infection inhibitor assay by mouse serum using immortalized MRC5 cells (pNifty cells). pNifty cells were seeded in 24-well plates at a concentration of 2.5 × 10^5^ cells per well, and after 12 h the cells were infected with pre-incubated HPIV3 virus (10^7^TCID_50_) with or without 5 μl mouse serum in 200 μl DMEM supplemented with 5% fetal bovine serum (FBS) and 1% penicillin and streptomycin (PS). At 4 h post-infection, the cells were washed and replaced in 200 μl of DMEM containing 10% FBS and 1% PS. At 48 h after medium change, the cells were washed with PBS and total RNA was extracted using the RNeasy Mini Kit (QIAGEN, Hilden, Germany) according to the manufacturer’s protocol. cDNA was synthesized using a cDNA synthesis kit (Toyobo, Osaka, Japan) and subjected to RT-PCR analysis with the SYBR Premix Ex gent Kit TaqII (Takara Bio) using an Applied Biosystems 7300 real-time PCR System. The primer sets used were as follows: HPIV3, 5′-CTCGAGGTTGTCAGGATATAG-3′ and 5′-CTTTGGGAGTTGAACACAGTT-3′; mGAPDH, 5′-CCATGGAGAAGGCTGGGG-3′ and 5′-CAAAGTTGTCATGGATGACC-3′.

## RESULTS

### GENERATION OF OML VACCINE AGAINST HPIV3-HN

**FIGURE 1:**
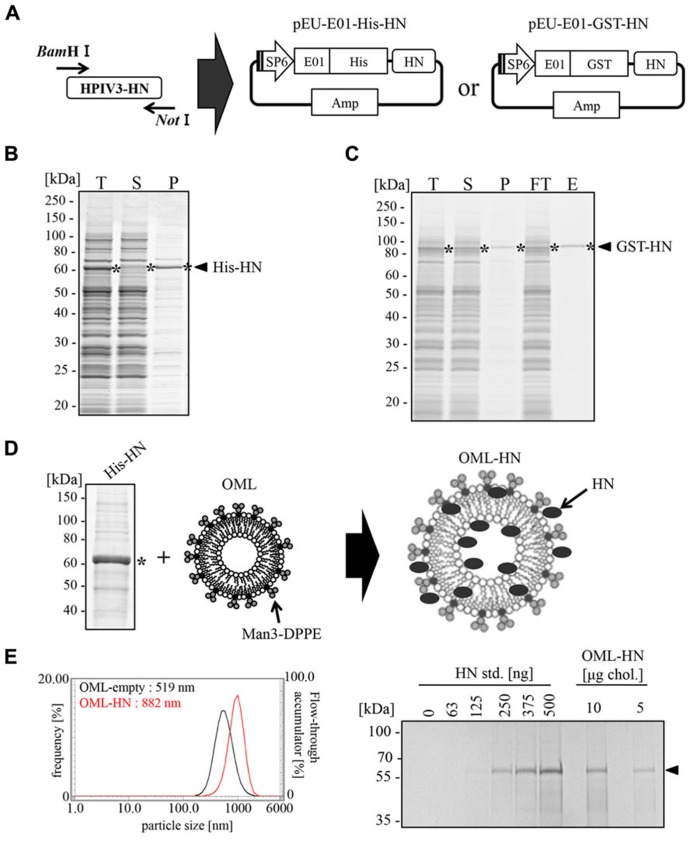
**Production of full-length HPIV3-HN and its encapsulation into OML.** **(A)** Schematic representation of the expression vector for the wheat germ cell-free system. Full-length HN cDNA was subcloned into either pEU-His or pEU-GST vectors. **(B)** Expression and solubility of His-HN proteins that were synthesized using the wheat germ cell-free system. The translation reaction mixture (T) was subjected to centrifu-gation followed by the separation of the soluble supernatant (S) and insoluble precipitate (P) fractions. These samples were subjected to SDS-PAGE and visualized with Coomassie Brilliant Blue (CBB) staining. The single asterisk indicates the band of His-HN. **(C)** Purification of recombinant GST-tagged HPIV3-HN proteins that were synthesized using the wheat germ cell-free system. The translation mixture (T) was subjected to the separation into supernatant (S) and precipitate (P) fractions by centrifugation. GST-HN proteins were purified using glutathione sepharose bead and then resolved by SDS-PAGE and CBB staining. The single asterisk indicates the band of GST-HN. FT, Flow through fraction; E, elute fraction. **(D)** Schematic of the production of OML-encapsulated His-HN. The single asterisk indicates the band of His-HN. **(E)** The amount of encapsulated HN was quantitated by SDS-PAGE (right panel). The diameter of OML-HN or control OML were measured by dynamic light scattering particle size analyzer (left panel). The arrow indicates the band of His-HN.

To produce the full-length HPIV3-HN antigen, we subcloned full-length HN cDNA into two different cell-free expression vectors, pEU-His and pEU-GST for the expression of N-terminally His-tagged or GST-tagged fusion proteins, respectively (**Figure 1A**). We found that both His-HN and GST-HN proteins were efficiently synthesized using the wheat germ cell-free system (**Figures 1B,C**). His-HN proteins precipitated into the insoluble fraction (**Figure 1B) were purified using Ni-sepharose beads in the presence of 8M Urea. GST-HN proteins were purified using glutathione sepharose beads in a regular buffer (**Figure 1C****).

After the large scale preparation of His-HN, the protein was incorporated into OML (**Figure 1D**). The particle diameter of HN-filling OML (OML-HN) and empty OML were 882 and 519 nm, respectively. The amount of carrier HN protein was approximately 32 mg per 1 mg cholesterol (**Figure 1E**) and the molar ratio of enclosed-OML to non-enclosed OML was found to be approximately 7:3 (data not shown).

### IMMUNIZATION OF MICE WITH OML-HN

**FIGURE 2:**
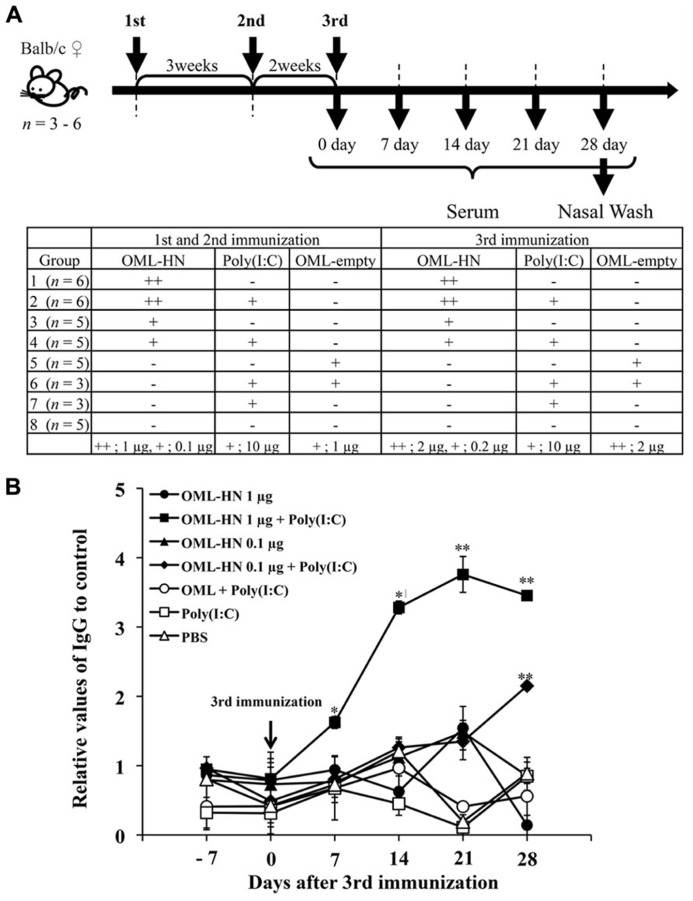
**Serum antibody responses in mice immunized with OML-HPIV3-HN.** **(A)** Balb/c mice were immunized with the indicated antigens and/or adjuvant on days 0, 21, 35 as described in the Materials and Methods. Serum was collected from each mouse on days 7, 14, 21, and 28 after the third immunization. The bottom panel summarizes the time course for the immunizations. **(B)** HN-specific IgG in pooled serum from each group was measured by ELISA. Data represents the mean ± SE. (*t* test: **P < 0.01, *P < 0.05)

We investigated whether intranasal administration of OML-HN could induce a humoral immune response against the HN. **Figure 2A** depicts the time course for the immunizations and blood collection from the immunized mice. Mice were immunized intranasally with OML-HN (1 or 0.1 μg) with or without Poly(I:C), OML with or without Poly(I:C), Poly(I:C) only, or PBS. One week after the third immunization, HN-specific serum IgG was detected in mice immunized with OML-HN (1 μg) plus Poly(I:C). The serum IgG levels were increased between days 7 and 14 and reached the peak at 21 days after the final immunization. Mice immunized with the lower amount of OML-HN (0.1 μg) plus Poly(I:C) also produced HN-specific serum IgG at 28 days after the last immunization (**Figure 2B**). In contrast, there was no significant HN-specific serum IgG in mice immunized with OML-HN without Poly(I:C) or the other negative control groups (**Figure 2B**).

**FIGURE 3:**
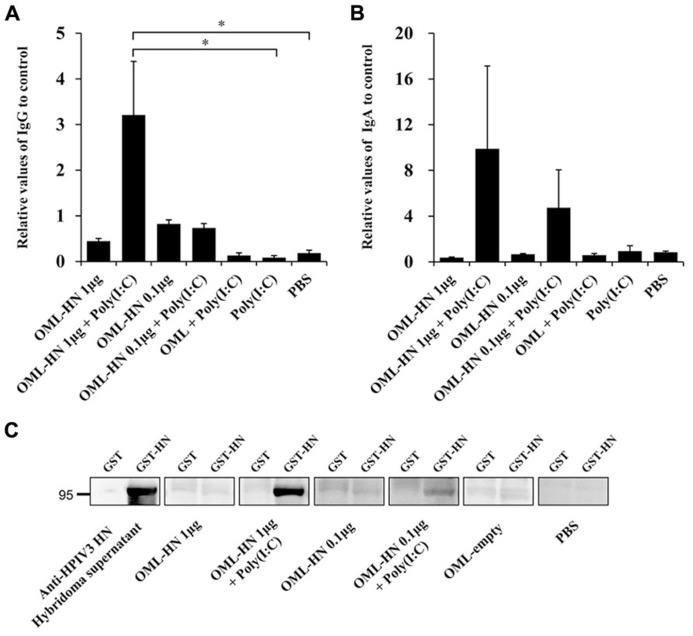
**Measurement of HPIV3-HN-specific serum IgG and nasal wash IgA.** **(A)** The levels of HN-specific IgG in the serum of each immunized mouse on day 21 after the third immunization were determined by ELISA. **(B)** The levels of HN-specific IgA in nasal wash fluid on day 28 after the third immunization were determined by ELISA. Each bar represents the mean ± SE (*t* test: *P < 0.05). **(C)** Immunoblot analysis of recombinant HN proteins in mouse sera. Recombinant GST or GST-HN proteins were subjected to SDS-PAGE followed by the immunoblotting with the indicated serum.

We next measured the levels of serum IgG and nasal wash IgA in each individual mouse by ELISA and immunoblotting (**Figures 3A–C**). Mice immunized with OML-HN (1 μg) plus Poly(I:C) exhibited prominent induction of HN-specific IgG (**Figures 3A,C**). HN-specific IgA in nasal wash fluid was most prominently induced in mice with OML-HN (1 μg) plus Poly(I:C) compared to other groups (**Figure 3B**). Interestingly, the induction of the HN-specific IgA was higher in mice that were immunized with the lower amount of antigens, OML-HN (0.1 μg) plus Poly(I:C) (**Figure 3B**).

### EPITOPE MAPPING OF INDUCED ANTIBODIES

**FIGURE 4:**
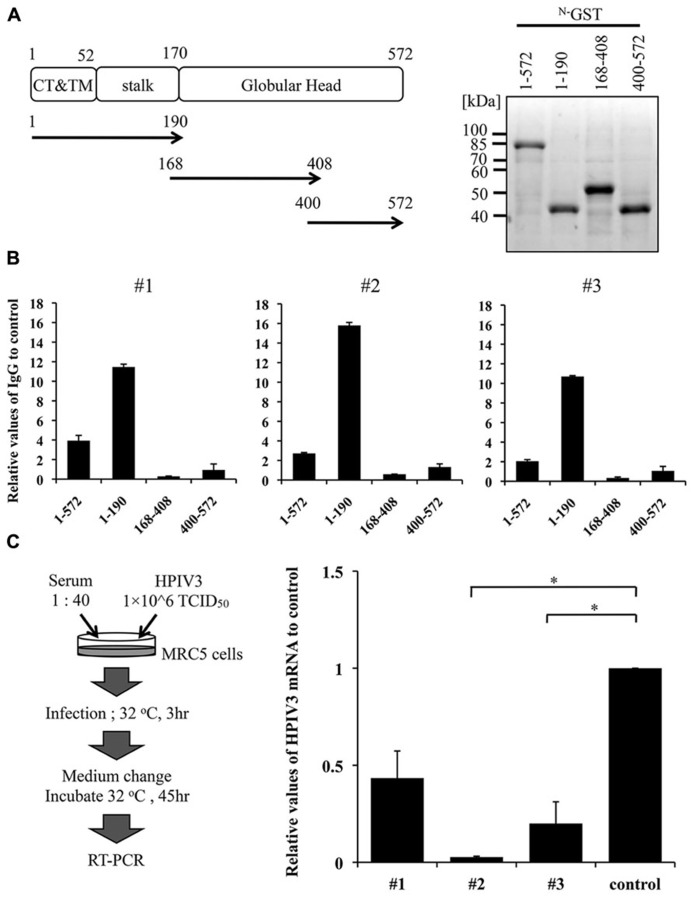
**Anti-infectious activity of mouse serum.** **(A,B)** Epitope mapping of antibodies induced in the immunized mouse. We selected arbitrary three representative sera from mice immunized with OML-HN (1 μg) plus Poly(I:C) that exhibited the highest HN-specific IgG induction (#1–#3). The full-length and three deletion mutants of GST-HN were produced using the wheat germ cell-free system. These purified proteins were separated by SDS-PAGE and visualized using CBB staining **(A)**. Using the recombinant HN proteins, the target region of the three sera (#1–#3) was analyzed by ELISA **(B)**. **(C)** Schematic representation of the experimental procedure of infection-inhibitory assay (left panel). Immunized mouse sera (#1–#3) were tested for this assay. An OML-empty-treated mouse serum was used as a control. The anti-infection ability was measured using quantitative real-time PCR for HPIV3-HN mRNA. Each bar represents the mean ± SE of two independent experiments as normalized by control serum (*t* test: *P < 0.05).

We next determined the region of HN that was recognized by the HN-specific serum IgG produced by the mice that were immunized with OML-HN (1 μg) plus Poly(I:C). Three domain mutants of HPIV3-HN, the N-terminal region (1-190), the middle region (168-408) and C-terminal region (400-572) were synthesized using the wheat germ cell-free system (**Figure 4A** left) and protein production was confirmed by SDS-PAGE (**Figure 4A** right). Based on ELISA analysis, all of the serum samples contained HN-specific antibodies that had high reactivity to the N-terminal region (**Figure 4B**).

### EFFECT OF OML-HN VACCINE ON HPIV3 INFECTION *IN VITRO*

We asked whether sera from the immunized mice could inhibit the HPIV3 infection of fibroblast cells. Infectious HPIV3 virions were pre-incubated with mouse serum harvested from three mice that were immunized with OML-HN plus Poly(I:C) and then used to infect MRC5 cells. Cellular HPIV3 mRNA was measured using quantitative reverse transcription PCR (qRT-PCR). The levels of HPIV3 mRNA were significantly reduced in cells pre-incubated with the immunized mouse serum compared to control serum that immunized with OML-empty (**Figure 4C**). These results indicate that the immunization with OML-HN plus Poly(I:C) induced serum antibodies that protect HPIV3 infection.

## DISCUSSION

Herein, we developed a novel subunit vaccine against HPIV3-HN using OML and a mucosal adjuvant Poly(I:C). Consequently we successfully induced antigen-specific immunoglobulin G and A with the immunization of lower quantities of HN antigen via the nasal route. Furthermore, the immunized mouse serum exhibited the ability to suppress the virus infection in cell culture model. These results indicate that our newly developed OML vaccine could offer a powerful means to protect HPIV3 infection.

In our current study, we used the wheat germ cell-free protein production system to synthesize full-length HN protein as an antigen (Takai et al., 2010). In comparison to cell-mediated procedures such as *Escherichia coli* and baculovirus systems, the wheat germ cell-free system is beneficial for the rapid and efficient preparation of high-quality proteins (Endo and Sawasaki, 2006). Moreover, this cell-free system is suitable for the generation of toxic viral proteins for immunization and beneficial for the purification of naturally folded proteins, as well as scalability. This system, however, may not be cost-effective for preparing large amounts of viral antigens for vaccine development. Therefore, efforts were made to reduce the amount of antigen needed vaccination. Herein, we utilized a OML and Poly(I:C) vaccination strategy in an attempt to reduce the amount of antigen required. OML is a lipid vesicle that has mannose on its surface, which aids in efficient targeting to APCs (Shimizu et al., 2007; Nishimura et al., 2013). In a previous report, antigenic proteins incorporated into OML were efficiently delivered to APCs via intranasal administration (Ishii and Kojima, 2010). In that report, intranasal administration of 5 μg ovalbumin (OVA) incorporated into OML four times effectively induced immune responses in mice (Ishii and Kojima, 2010). Poly(I:C) is a synthetic double-stranded RNA (dsRNA) molecule that induces effective mucosal immune responses by stimulating Toll-like receptor 3 (TLR3) as a molecular mimic of dsRNA, which is a byproduct of viral replication (Ichinohe et al., 2005; Hasegawa et al., 2009). The efficacy of nasal vaccines made of subunit proteins in the combination with mucosal adjuvants was demonstrated for influenza virus and RSV (Ichinohe et al., 2005; Hasegawa et al., 2009; Ainai et al., 2010; Kamphuis et al., 2013). We utilized a mucosal adjuvant Poly(I:C) to induce HN-specific antibodies in serum and nasal wash fluid through intranasal immunization with OML-HN. Using our vaccination strategy, we were able to decrease the amount of antigen required to 20% relative to previous reports (Mader et al., 2000; Ishii and Kojima, 2010).

The mucosa of respiratory tracts is the site of defense against virus infection since respiratory viruses attack and infect the respiratory mucosal tissues and cells (Tamura and Kurata, 2004). Mucosa is generally protected by mucin and defensin produced from goblet cells and Paneth cells. The TLR family members, TLR3, TLR7, TLR8, and TLR9 can recognize viral nucleotides and induces type I interferon (IFN-I) production if viruses intrude into tissues beyond the barrier. IFN-I activates the defense mechanism against virus by promoting the maturation of DCs and the induction of NK cells (Takeda et al., 2003; Akira et al., 2006). On the other hand, it is known that Microfold cells (M cells) promotes adherence and transport of antigens to APCs (Sato and Kiyono, 2012). M cells reside in the follicle-associated epithelium of Peyer’s patches in the intestinal tract or nasal lymphoid tissue (NALT) of rodents in the upper respiratory tract, and plays a pivotal role in the induction of antigen-specific immunity (Nochi and Kiyono, 2006). The APCs promote adaptive immune responses by presenting antigens to naïve B cells and activate it to differentiate into antigen specific B cells. In the mucosa, secretory IgA is transported to mucosal surface by polymeric Ig receptor (pIgR) and the secreted IgA plays an important role in the protection of viral infection in the respiratory tract (Mostov and Deitcher, 1986). It is known that the intranasal immunization can activate mucosal immunity thereby enhancing the induction of mucosal IgA in addition to the generation of systemic IgG against viral antigen. Our current study employed OML as an effective tool to deliver the antigen to APCs and M cells in respiratory mucosa. A recent report demonstrated that OML-mediated intranasal immunization can efficiently induce Th2 cytokines such as IL-5 and IL-6 that eventually help produce secretory IgA in mucosal system in mouse model (Ishii and Kojima, 2010). We further combined OML with a mucosal adjuvant Poly(I:C) to facilitate the specific mucosal immunity against HPIV3-HN. Poly(I:C) has been shown to be an effective mucosal adjuvant stimulating TLR3 as a molecular mimic. A previous report indicated that a nasal influenza virus vaccine combined with Poly(I:C) synergistically induced IFN-1 and Th2 cytokine leading to an effective humoral immunity including secretory IgA in mucosa (Ichinohe et al., 2005). Our current study also demonstrated that the combinatory use of nasal vaccine with Poly(I:C) has a profound effect in inducing mucosal immunity against viral antigen.

Our newly developed OML-HN vaccine has several advantages as compared with previously developed vaccine methods including live attenuated vaccines. Although there is no practical prophylactic vaccine against HPIV3 infection, several previous studies have indicated that attenuated vaccines created by reducing the virulence of HPIV3 can indeed effectively induce the mucosal immunity when treated by intranasal administration (Karron et al., 2003). However, a major problem of these vaccines is their potential to cause a live infection in infants and immunocompromised hosts. Furthermore, there is a small risk of reversion to virulence by genetic mutations that results in the onset of severe disease. Therefore, it is desirable to develop a safer HPIV3 vaccine with lower risks of infection. On the other hand, intranasal subunit vaccines against HPIV3 have been demonstrated to be effective in animal models without the risk of viral replication and live infection. According to a report by Ray et al., the intranasal administration of HN and F proteins extracted from virions could induce significant anti-viral immunity in hamsters (Ray et al., 1988). However a drawback of subunit vaccines is their requirements for large amounts of antigens and concomitant high cost. Therefore, it is important to develop a cost-effective subunit vaccine that dispenses with substantial quantity of antigens. In order to overcome this problem, we used OML and Poly (I:C) aiming for efficient vaccine delivery and immune response, respectively. Indeed, our current study demonstrated that a combination of OML-HN with poly(I:C) induced antigen-specific IgA and IgG by three times more than the immunization without poly(I:C). The safety in the use of either OML or poly(I:C) has been reported in previous studies. For OML-based vaccine, Fukasawa et al. have reported that OML has indeed no obvious toxicity and immunogenicity by itself (Fukasawa et al., 1998). Furthermore, Poly(I:C) has shown to be non-toxic as compared with conventional vaccine adjuvant such as cholera toxin subunit B (Ichinohe et al., 2005). However, further careful analysis should be necessary to validate the effectiveness and feasibility of our newly-developed vaccine strategy using virus infection models with multiple genotypes of HPIV3.

In our current study, we demonstrated that the OML-based vaccine incorporated with full-length HN protein induced IgG that targets the N-terminal region of HN protein. The N-terminal region of HN contains the stalk region while the C-terminal region contains the globular head domain. The stalk region of HN is known to play a crucial role in virion-host cell fusion via an interaction with F protein while the globular head binds sialic acid and neuraminidase (Moscona, 2005; Porotto et al., 2012). Although effective antigenic epitopes for HPIV vaccine remain elusive (Henrickson, 2003), a monoclonal antibody targeting the stalk region of HPIV2-HN has been shown to have a profound inhibitory activity against viral infection (Yuasa et al., 1995). Based on the observation, it seems that the antibodies induced by our vaccine system could also target the stalk region since they effectively blocked the viral infection in cell culture model. Further careful analysis will be required for the mapping of the epitope affecting virus infection in our current model.

In this study, we did not investigate other routes of antigen administration besides the intranasal route. However, previous studies have indicated that non-nasal immunization of HPIV3 components failed to prohibit the infection of HPIV3 in a cotton rat model. Indeed, the intramuscular immunization with HN and F recombinant proteins could not protect virus infection in upper respiratory tract although it had some effects on the protection of pneumonia and lower respiratory tract infection (Ambrose et al., 1991). It is generally believed that intranasal immunization has a great benefit for protecting virus infection itself by inducing antigen-specific secretory IgA in respiratory mucosa (Hirabayashi et al., 1990; Durrer et al., 2003). Our current study also confirmed this advantage of nasal vaccination where the OML-based nasal vaccine provides high performance for the induction of antigen-specific secretory IgA in nasal wash fluids. Therefore, intranasal administration of OML-based vaccine with poly(I:C) adjuvant could be an effective way of vaccination against respiratory viruses including HPIV3.

## Conflict of Interest Statement

The authors, editor, and chief editor declare that while the authors Kyosuke Senchi, Satoko Matsunaga, Akihide Ryo, and the review editor Ichiro Aoki are currently employed by the same institution (Yokohama City University School of Medicine, Japan) and while Hirokazu Kimura and Hironori Sato are currently employed by the same institution (National Institute of Infectious Diseases, Japan) there has been no conflict of interest during the review and handling of this manuscript.
